# High Dosages of Equine Chorionic Gonadotropin Exert Adverse Effects on the Developmental Competence of IVF-Derived Mouse Embryos and Cause Oxidative Stress-Induced Aneuploidy

**DOI:** 10.3389/fcell.2020.609290

**Published:** 2021-02-09

**Authors:** En Lin, Zhiling Li, Yue Huang, Gaizhen Ru, Pei He

**Affiliations:** Department of Reproductive Center, The First Affiliated Hospital of Shantou University Medical College, Shantou University, Shantou, China

**Keywords:** equine chorionic gonadotropin, aneuploidy, reactive oxygen species, DNA damage response, spindle assembly checkpoint, MAD2L1, Aurora B

## Abstract

Gonadotropins play vital roles in the regulation of female reproductive ability and fertility. Our study aimed to determine the effects of superovulation induced by increasing doses of equine chorionic gonadotropin [eCG; also referred to as pregnant mare serum gonadotropin (PMSG)] on the developmental competence of mouse embryos and on aneuploidy formation during *in vitro* fertilization (IVF). eCG dose-dependently enhanced the oocyte yield from each mouse. Administration of 15 IU eCG significantly reduced the fertilization rate and the formation of four-cell embryos and blastocysts and increased the risk of chromosome aneuploidy. The IVF-derived blastocysts in the 15 IU eCG treatment group had the fewest total cells, inner cell mass (ICM) cells and trophectoderm (TE) cells. Moreover, more blastocysts and fewer apoptotic cells were observed in the 0, 5, and 10 IU eCG treatment groups than in the 15 IU eCG treatment group. We also investigated reactive oxygen species (ROS) levels and variations in several variables: mitochondrial membrane potential (MMP); active mitochondria; mitochondrial superoxide production; adenosine triphosphate (ATP) content; spindle structures; chromosome karyotypes; microfilament distribution; and the expression of Aurora B [an important component of the chromosomal passenger complex (CPC)], the spindle assembly checkpoint (SAC) protein mitotic arrest deficient 2 like 1 (MAD2L1), and the DNA damage response (DDR) protein γH2AX. Injection of 15 IU eCG increased ROS levels, rapidly reduced MMP, increased active mitochondria numbers and mitochondrial superoxide production, reduced ATP content, increased abnormal spindle formation rates, and induced abnormalities in chromosome number and microfilament distribution, suggesting that a high dose of eCG might alter developmental competence and exert negative effects on IVF-obtained mouse embryos. Additionally, the appearance of γH2AX and the significantly increased expression of Aurora B and MAD2L1 suggested that administration of relatively high doses of eCG caused Aurora B-mediated SAC activation triggered by ROS-induced DNA damage in early mouse IVF-derived embryos for self-correction of aneuploidy formation. These findings improve our understanding of the application of gonadotropins and provide a theoretical basis for gonadotropin treatment.

## Introduction

The use of gonadotropins is currently a considerable part of assisted reproductive technology (ART) because it enables procurement of large numbers of oocytes from a single *in vitro* fertilization (IVF) cycle (Zolbin et al., 2018). However, the side effects of ovulation induction treatments, such as ovarian hyperstimulation syndrome (OHSS) and the potential for increased long-term ovarian cancer risk (Farhud et al., 2019) are raising concerns regarding the safety of ovulation stimulants to the mother. Whether high doses of gonadotropins impact the fertilization potential of oocytes and the further developmental quality of the embryos is a subject of concern (Anderson et al., 2018; Wu et al., 2018). Increases in the levels of endogenous gonadotropins (e.g., due to the reduction in the ovarian reserve in aged females) or exogenous gonadotropins (e.g., due to ovulation stimulation in ART) are potential mechanisms of aneuploidy, a major factor in early fetal death and severe intellectual disability (Dursun et al., 2006). Additionally, hormonal stimulation can independently lead to cognitive dysplasia (Rumbold et al., 2017), and a high-quality study has shown that ovulation induction in the absence of ART increases the risk of developmental disorders in children (Bay et al., 2013). Furthermore, ovarian hyperstimulation might negatively affect cardiac metabolic outcomes in IVF offspring by altering the early environment of the oocytes and/or embryos, thus leading to epigenetic modifications of pivotal metabolic systems implicated in blood pressure regulation (La Bastide-Van Gemert et al., 2014; Seggers et al., 2014). Superovulated human oocytes show hypermethylation of H19 and demethylation at paternally expressed gene 1 (PEG1) (Sato et al., 2007), indicating that superovulation might lead to false imprinting (Lawrence and Moley, 2008; Calicchio et al., 2014).

Superovulation using equine chorionic gonadotropin (eCG) and human chorionic gonadotropin (HCG) in place of follicle-stimulating hormone (FSH) and luteinizing hormone (LH) is a widely used protocol for maximizing the numbers of oocytes and preimplantation embryos obtained from animals, respectively (Uysal et al., 2018). Results from animal studies have shown that gonadotropin stimulation can reduce the developmental potential of embryos *in vitro* (Karagenc et al., 2004; Wu et al., 2013; Swann, 2014), cause mitochondrial dysfunction in mouse oocytes (Ge et al., 2012), alter DNA methyltransferase protein expression (Uysal et al., 2018), affect the translational control of maternally stored messenger RNA (mRNA) (Ozturk et al., 2016) in early embryos, impair embryo quality and the uterine milieu (Ertzeid and Storeng, 2001), and change the appearance of imprinted genes in the midgestation mouse placenta (Fortier et al., 2008), resulting in delayed development (Ertzeid and Storeng, 1992; Ingrid and Thomas, 2001) or embryo death (Beaumont and Smith, 1975) and leading to an increased rate of offspring malformations (Sakai and Endo, 1987). Moreover, anti-eCG serum can effectively reduce the negative effect of eCG on the normal ovulation of mice (Lin et al., 2015).

Increasing amounts of evidence are showing that reactive oxygen species (ROS) play a crucial role in the regulation of oocyte maturation and fertilization (Lopes et al., 2010; Ge et al., 2012). Oocyte development and maturation depend on a dynamic balance between oxidant and antioxidant production (Kala et al., 2016; Han et al., 2017). Regardless of the sources of superoxide anion free radicals in female germ cells, when oxidative stimulation is well-balanced and suitable for physiological processes (e.g., zona pellucida hardening and oocyte maturation), H_2_O_2_ produced by disproportionation exerts positive effects. However, the overproduction of ROS is associated with negative outcomes (e.g., impaired mitochondrial function, aging, DNA damage and impaired chromosome segregation; Kala et al., 2016; Aitken, 2020). Mitochondrial dysfunction is linked to non-physiological ovarian stimulation and *in vitro* maturation (Ge et al., 2012). Mitochondria are the primary cellular sources of ROS, and these organelles are vulnerable to ROS-induced damage (Yamada-Fukunaga et al., 2013). ROS can disrupt the flow of electrons in the mitochondria and promote electron leakage and ROS production, thereby enhancing the generation of ROS in the positive feedback system. Because of this chemical reaction, oxidative stress persists and has a considerable effect on apoptosis (Aitken, 2020). Moreover, because eCG has a circulating half-life of 40–125 h, its residues often affect follicular maturation and ovulation and thereby induce persistent estrogen secretion from large anovulatory follicles, which might lead to supraphysiological levels of estrogen (Lin et al., 2015). It has been proposed that estrogen can generate ROS both directly by acting on mitochondria (Felty et al., 2005) and indirectly through IL-1β and TNF-α (Roy et al., 2007). Additionally, the exceedingly high estradiol concentrations induced by ovarian stimulation during IVF increase extramitochondrial ROS production in endometrial epithelial cells, which leads to mitochondrial dysfunction (Chou et al., 2020).

Aurora B kinase (Aurora B/AURKB) is an element of the chromosomal passenger complex (CPC) as well as a serine/threonine kinase that is well-known for its important functions in multiple events during mitosis, including sister chromosome adhesion, chromosome condensation, chromosome biorientation, kinetochore-microtubule (KT-MT) attachments, spindle assembly checkpoint (SAC) activation, cytokinesis, and chromosome segregation (Honma et al., 2014; Huang et al., 2019; Li et al., 2019; Zhang et al., 2020). Germ cells that undergo meiosis also express an Aurora B homolog named Aurora C, which can play a role in the CPC as well (Ruchaud et al., 2007). During meiosis in mouse oocytes, Aurora B is directly involved in SAC function *via* the kinetochore recruitment of SAC components, while Aurora C is thought to play an indirect role in SAC activation through depolymerization of improper KT-MT attachments (Balboula and Schindler, 2014; Nguyen et al., 2014). In mitosis, among other functions, Aurora B directly participates in the maintenance of SAC activation by recruiting key factors to kinetochores and indirectly invokes SAC signaling by destabilizing incorrect KT-MT attachments (Ruchaud et al., 2007; Saurin et al., 2011; Balboula and Schindler, 2014). However, it is important to note that Aurora C is not inimitably essential for SAC activation or cytokinesis, and these events may be Aurora B specific (Yasui et al., 2004; Balboula and Schindler, 2014). Nguyen et al. (2018) described a unique requirement for Aurora B to negatively regulate Aurora C to prevent aneuploidy. Only Aurora B overexpression (not Aurora C overexpression) can rescue chromosome alignment defects (Shuda et al., 2009). Aurora B has been reported to potentiate Mps1 (also known as TTK) activation to quickly establish the mitotic checkpoint (Saurin et al., 2011); moreover, our previous findings have shown that mitotic arrest deficient 2 like 1 (MAD2L1; also referred to as MAD2) recruitment to kinetochores by TTK occurs at the onset of oxidative damage in embryos during SAC activation (Wu et al., 2017). In addition, MAD2 prevents chromosome segregation defects and maintains chromosome stability through modulation of the mitotic functions of Aurora B (Shandilya et al., 2016). In a previous study, we found that ROS-induced DNA damage triggers SAC activation *via* Aurora B to hinder aneuploidy in early IVF-derived mouse embryos and that Aurora B is an important regulator of mitosis that assists in self-correction when chromosomal abnormalities occur during embryo development (Li et al., 2019). Hence, Aurora C will not be discussed further in this article. The current study focused on the role of the critical CPC kinase Aurora B during mitosis in early IVF-derived mouse embryos. Our results have consistently shown that oxidative stress increases the probability of sex chromosome aneuploidy in male mouse embryos by inducing chromosome mis-segregation and inhibits the expression levels of male reproduction-related proteins; these effects might negatively affect the reproductive health of IVF-obtained male offspring (Huang et al., 2019). We consequently hypothesized that administration of high doses of eCG would affect the viability and development of IVF-derived mouse embryos and cause oxidative stress-induced aneuploidy, thereby triggering Aurora B-mediated SAC activation.

## Materials and Methods

### Experimental Animals

Adult Kunming mice (male: aged 3–6 months; female: aged 6–8 weeks) were ordered from the animal center at Shantou University Medical College. The mice were randomly assigned to various groups. All work strictly abided by the International Guiding Principles for Biomedical Research Involving Animals (2012 edition) published by the Council for the International Organization of Medical Sciences, and all experimental protocols were approved by the Institutional Animal Care and Use Committee (IACUC) of Shantou University Medical College (SUMC2018-049).

### Sperm Capacitation, Oocyte Collection, IVF, and Embryo/Blastocyst Culture

As described previously (Huang et al., 2019; Li et al., 2019), prior to oocyte collection, sperm were removed from the epididymal tails of male mice, moved to capacitation medium prepared in advance with human tubal fluid (HTF) medium (Cooper Surgical, USA) containing 1.5% bovine serum albumin (BSA) and immediately incubated in an incubator (37°C, 5% CO_2_) for 60 min. Adult female mice were intraperitoneally injected with 0, 5, 10, or 15 IU eCG (Ningbo Second Hormone Factory, China); 48 h later, the mice received an injection of 10 IU HCG (Ningbo Second Hormone Factory, China). A dose of 10 IU eCG is the most commonly used dose for ovulation induction in female Kunming mice (Wu et al., 2016; Huang et al., 2019; Li et al., 2019). Therefore, we decided to use up to 15 IU eCG (a high dose of eCG) in our experiments. When injected, eCG or HCG was diluted with 0.9% NaCl solution. The same amount of 0.9% NaCl solution was given to the mice in the control (0 IU eCG) group. Superovulated but unmated female mice were euthanized at 13–15 h following HCG injection. Cumulus-oocyte complexes were collected from the ovaries and transferred to droplets, which were prepared by adding capacitated sperm to an HTF solution containing 0.4% BSA; the complex-containing droplets were then incubated in 5% CO_2_ at 37°C for 6 h. After fertilization, the zygotes were washed three times in HTF medium at 37°C and cultured in new embryo culture medium (HTF medium with both 0.4% BSA and 10% fetal bovine serum) at 37°C under an atmosphere with 5% CO_2_. For blastocyst culture, we used the same protocol described above but with blastocyst culture medium (Cooper Surgical, USA) instead of embryo culture medium.

### Determination of Cytoplasmic ROS Products

DCFH-DA (Sigma, USA) is a cell-permeable fluorogenic probe useful for determining the degree of overall oxidative stress. The stock solution of DCFH-DA was diluted with HTF medium to a final concentration of 10 μmol/L. Zygotes from each treatment group were transferred into DCFH-DA droplets, incubated at 37°C for 30 min, washed three times in phosphate-buffered saline (PBS) and mounted on adhesive microscope slides. The glass slides were observed by fluorescence microscopy (Nikon Eclipse 90 Ni-E, Japan). The fluorescence intensity (FI) was detected *via* Image-Pro Plus 6.0 software (Media Cybernetics, USA).

### Evaluation of Mitochondrial Membrane Potential (MMP)

JC-1 (Sigma, USA) is a type of cationic fluorescent membrane-permeable carbocyanine dye that can be used as a ratiometric indicator of MMP in zygotes. Mitochondria with high and low membrane potentials are stained red and green, respectively, by this probe. A 5 mg/mL stock solution was prepared in dimethyl sulfoxide (DMSO), and this solution was diluted with PBS to a 1.25 μmol/L working solution. Zygotes were incubated for 30 min at 37°C in dye-containing medium and washed three times with PBS (3 min per wash) prior to experimental observation. Under an upright fluorescence microscope (Nikon Eclipse Ni-E, Japan), the J-monomer (green) was observed selectively with a 488-nm argon-ion laser source. The J-aggregate (red) was observed with a 568-nm argon-krypton laser line. The red/green FI in zygotes was detected using Image-Pro Plus 6.0 software.

### Detection of Active Mitochondria and Superoxide Anion Production

MitoTracker™ Green FM (Invitrogen, USA) is a green fluorescent dye that stains mitochondria in embryos and accumulates in active mitochondria, and its accumulation is independent of the membrane potential. A small amount of 1 mM MitoTracker Green stock solution was added to HTF culture medium at a ratio of 1:5000–1:50000 to obtain a final concentration in the range of 20–200 nM. A 5 mM mitochondrial superoxide indicator MitoSOX™ Red (Invitrogen, USA) stock solution was then diluted in HTF culture medium to obtain a 5 μM MitoSOX Red working solution. One hundred microliters of MitoTracker Green or MitoSOX Red working solution was then applied to the embryos. The embryos were then incubated for 30 min at 37°C in the dark, gently washed three times with warm buffer and mounted in warm buffer on adhesive microscope slides for imaging. The fluorescence signal (FI value) for each embryo was measured with Image-Pro Plus 6.0 software.

### Determination of the Adenosine Triphosphate (ATP) Content

The amount of ATP present in embryos was quantified using a CellTiter-Glo® 2.0 Assay (Promega, Madison, WI, USA) with a multimode microplate reader (Synergy H1, BioTek, USA). rATP (10 mM, Promega, USA) was used to generate a 14-point ATP standard curve for each analysis before addition of CellTiter-Glo® 2.0 reagent in the same 96-well plate in which samples were examined. The ATP content of each group was calculated with the formula obtained from linear regression of the standard curve. All embryos treated with increasing amounts of eCG were analyzed simultaneously to reduce any potential variability.

### Immunofluorescence and DAPI Staining

The protocol used for the immunofluorescence staining of embryos was previously described by our research group (Huang et al., 2019; Li et al., 2019). The sections were visualized using an LSM 800 laser scanning microscope (ZEISS, Germany). F-actin was labeled with Alexa Fluor™ 488 phalloidin, which was purchased from Invitrogen (Carlsbad, CA, USA). A rabbit anti-gamma H2A.X antibody (phospho-S139), a rabbit anti-Aurora B antibody, DAPI staining solution and a goat anti-rabbit IgG H&L antibody (Alexa Fluor® 488) were obtained from Abcam (Cambridge, UK). An Alexa Fluor® 594-conjugated mouse anti-MAD2 antibody was obtained from Santa Cruz (Dallas, TX, USA). A mouse anti-α-tubulin-FITC monoclonal antibody was procured from Sigma-Aldrich (St. Louis, MO, USA).

### Fluorescence *in situ* Hybridization (FISH)

RNA FISH probe mixes for hybridization to Aurora B (FAM-conjugated) and MAD2L1 (Cy3-conjugated) mRNA molecules were designed and synthesized by GenePharma (Shanghai, China). FISH assays were performed manually with an RNA FISH kit (GenePharma) following the manufacturer's recommended protocol. Embryos mounted on polylysine slides were fixed with 4% paraformaldehyde for 15 min, transferred to 0.1% Triton X-100 for 15 min, subjected to two 5-min rinses in PBS and treated sequentially with 2× saline sodium citrate (SSC; 30 min at 37°C), 70% ethanol (3 min at room temperature), 85% ethanol (3 min at room temperature), and anhydrous ethanol (3 min at room temperature) to enable RNA probe access. Target probe mixes for Aurora B and MAD2L1, diethyl pyrocarbonate (DEPC)-treated water and warm hybridization solution were added onto the slides, and the slides were then incubated in the dark for 5 min at 73°C for denaturation and in a humid atmosphere for 12–16 h at 37°C to allow the probe to hybridize with the RNA targets. The slides were subsequently washed with 0.1% Tween-20 for 5 min and incubated with two SSC solutions: 2× SSC (5 min at room temperature) and 1× SSC (5 min at room temperature). Chromatin was then stained with DAPI, and the slides were subjected to two 5-min washes in PBS. The Aurora B and MAD2L1 mRNA fluorescence signals were imaged under an LSM 800 laser-scanning microscope (ZEISS, Germany).

### Karyotype Analysis

Typical protocols from our previous studies (Huang et al., 2019; Li et al., 2019) were used and are summarized below. Briefly, karyotype analysis of mouse embryos is a four-step procedure. In the first step, the embryos were added to HTF culture medium with 0.05 μg/mL podophyllotoxin and 0.05 μg/mL vinblastine and incubated for 12–16 h. Podophyllotoxin damages the microfilament system to prevent prokaryotic fusion, and vincristine interferes with tubulin polymerization, blocks spindle formation, and maintains embryos at the mid-cleavage stage. Subsequently, the embryos were immersed in 0.1% pancreatin to remove the zona pellucidae and mounted on adhesive slides. Ultrapure water containing 20% fetal bovine serum was mixed with a 0.9% sodium citrate solution at a ratio of 1:6 to form a hypotonic solution. The slides with the embryo samples were added to the preheated hypotonic solution, placed in an incubator at 37°C for 30 min, and then incubated with a series of conventional fixative solutions: fixative I (methanol:glacial acetic acid:ultrapure water = 15:3:1, 5 min at room temperature), fixative II (methanol:glacial acetic acid = 3:1, overnight at room temperature), and fixative III (methanol:glacial acetic acid:ultrapure water = 3:3:1, 1 min at room temperature). In the last step, the slides were stained in Giemsa staining solution for 30 min, washed with ultrapure water and dried in air. To prevent confusion, only one embryo was lysed after exposure to the fixative solutions and slowly “melted” onto one clean microscope slide. The slides were then scanned using a standard bright-field microscope (Nikon Eclipse 90 Ni-E).

### Terminal Deoxynucleotidyl Transferase-Mediated dUTP Nick-End Labeling (TUNEL) Assay

A RiboAPO^TM^ One-Step TUNEL Apoptosis Detection Kit (Red) (RiboBio, China) was used to detect blastocyst apoptosis. The level of apoptosis and the blastocyst internal population of cells, which is termed the inner cell mass (ICM), were evaluated through a TUNEL assay and immunofluorescence staining of octamer-binding transcription factor 4 (Oct4), respectively. The apoptosis rate is expressed as the percentage of TUNEL-positive cells relative to the total number of cells (DAPI-stained cells) in a single blastocyst. The total cell number (as indicated by DAPI staining) in each blastocyst minus the ICM (as indicated by Oct4 staining) was calculated as the number of trophectoderm (TE) cells, and the ICM/TE cell ratio was then calculated. The cell counting method was described by Carstea et al. (2012). A rabbit anti-oct4 antibody and a goat anti-rabbit IgG H&L antibody (Alexa Fluor® 488), which were purchased from Abcam (Cambridge, UK), were used as the primary and secondary antibodies, respectively.

### Real-Time Quantitative PCR (RT-PCR)

Approximately 150 zygotes were prepared, and RNA was extracted with an RNAprep Pure Micro Kit (Tiangen, China). High-quality RNA was used for cDNA synthesis with FastKing gDNA Dispelling RT SuperMix (Tiangen, China). The C_t_ values were determined by RT-PCR using a CFX Connect^TM^ Real-Time PCR Detection System (Bio-Rad, USA) and Talent qPCR PreMix (SYBR Green) (Tiangen, China). The expression of β-actin was used as an internal control. The relative mRNA expression level of each target gene is represented as the fold change calculated using the 2^−ΔΔCt^ method.

### Protein Extraction From Zygotes

Lysates of 150 zygotes from each treatment group were prepared *via* addition of ice-cold RIPA lysis buffer (Beyotime, China) containing a protease and phosphatase inhibitor cocktail (Beyotime, China) to a final volume of 10 μL. A histone extraction kit (Abcam, UK) was used to extract histone proteins from zygotes. The protein concentrations were quantified using a BCA kit (Boster, China) with BSA as the standard.

### Automated Western Immunoblotting

All primary rabbit antibodies used in automated capillary western immunoblotting were obtained from Abcam (Cambridge, UK). All dilutions were prepared in Antibody Diluent II buffer (ProteinSimple, USA). The anti-rabbit secondary antibody (ProteinSimple, USA) was ready-to-use without any handling. Automated western immunoblotting was performed using Wes Simple^TM^ (ProteinSimple, USA) in accordance with the manufacturer's instructions (Nelson et al., 2017; Sage et al., 2020).

### Statistical Analysis

The results from at least three independent experiments were collected and analyzed with SPSS version 19.0 software (IBM, Armonk, NY, USA). The data are presented as the means ± standard deviations (*SD*s) and were assessed with Student's *t*-tests and ANOVA. Some values are expressed as percentages and were compared using the Pearson's chi-square test or Fisher's exact test. In all the statistical tests, differences were considered significant if *P* < 0.05.

## Results

### Effects of Different eCG Doses on the Numbers of Oocytes Obtained From Mice

**Figure 1:**
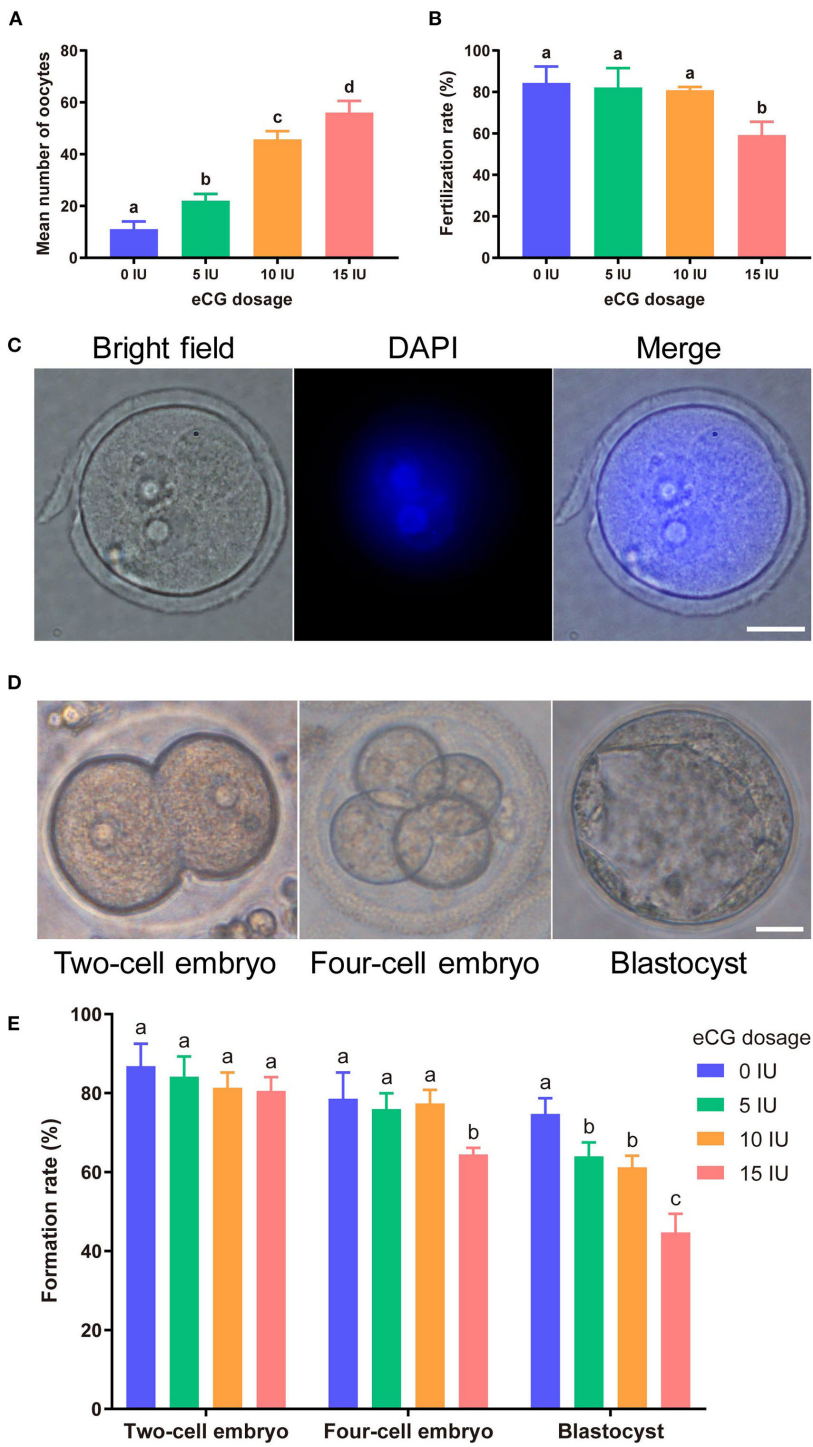
Effects of different eCG doses on the development of IVF-derived mouse embryos. **(A)** Mean numbers (means ± *SD*s) of oocytes obtained after administration of 0, 5, 10, and 15 IU eCG (with 10 IU HCG injection). ^a,b,c,d^Bars labeled with different letters are significantly different from each other (*P* < 0.05). **(B)** Fertilization rates after treatments with different eCG doses. ^a,b^Bars labeled with different superscript letters are significantly different from each other (*P* < 0.05). **(C)** Representative images of 2PN zygotes in mouse embryos at 8–10 h post insemination (hpi) (scale bar = 20 μm). **(D)** Morphological appearances of a two-cell embryo, a four-cell mouse embryo and a single blastocyst. Scale bar = 20 μm. **(E)** Rates of two-cell embryo, four-cell embryo and blastocyst formation with different doses of eCG. The data are expressed as the means ± *SD*s. ^a,b,c^Columns within a group marked with different superscript letters are significantly different from each other, *P* < 0.05.

Increasing eCG doses (with 10 IU HCG injection) markedly and dose-dependently enhanced mouse oocyte yield. The mean numbers of oocytes retrieved from female Kunming mice after injections of 0, 5, 10, and 15 IU eCG were 11.00 ± 3.00, 22.00 ± 2.65, 45.67 ± 3.22, and 56.00 ± 4.58, respectively. The numbers of mouse oocytes increased with increasing doses of eCG (*P* < 0.05) (Figure 1A).

### Effects of Different eCG Doses on the Development of IVF-Derived Mouse Embryos

To identify the effects of different eCG doses on the developmental potential, oocytes obtained after ovulation were fertilized and cultured *in vitro*. At 8–10 hpi, two-pronuclear (2PN) zygotes and/or the second polar body could be observed after the successful fertilization of normal mouse zygotes (Figure 1C). Nearly identical fertilization rates were found for the zygotes in the control (0 IU eCG injection) group (84.38 ± 7.94), 5 IU eCG injection group (82.23 ± 9.29) and 10 IU eCG injection group (80.91 ± 1.58) (*P* > 0.05). Compared with the experimental groups that received 0, 5, and 10 IU eCG, the group that received 15 IU eCG (59.22 ± 6.39) exhibited at significantly lower fertilization rate (*P* < 0.05) (Figure 1B). The rates of two-cell embryo formation in the groups of mice administered 0, 5, 10, and 15 IU eCG were 86.87 ± 5.65, 84.13 ± 5.16, 81.35 ± 3.87, and 80.54 ± 3.50, respectively. Increasing the eCG dose had no significant effect on the rate of two-cell embryo formation (*P* > 0.05). The rate of four-cell embryo formation was significantly lower in the 15 IU eCG (64.45 ± 1.72) treatment group than in the 0 IU eCG (78.60 ± 6.59), 5 IU eCG (75.91 ± 4.03), and 10 IU eCG (77.36 ± 3.46) treatment groups (*P* < 0.05). Similarly, administration of 15 IU eCG (44.68 ± 4.72) significantly reduced the rate of blastocyst formation (*P* < 0.05). Furthermore, the rate of blastocyst formation in the control group (74.72 ± 4.00) was higher than those in the 5, 10, and 15 IU eCG groups, but no significant difference was found between the 5 IU eCG (63.99 ± 3.53) and 10 IU eCG (61.21 ± 2.90) treatment groups (Figures 1D,E).

### Effects of Different eCG Doses on ROS Concentrations in IVF-Derived Mouse Embryos

**Figure 2:**
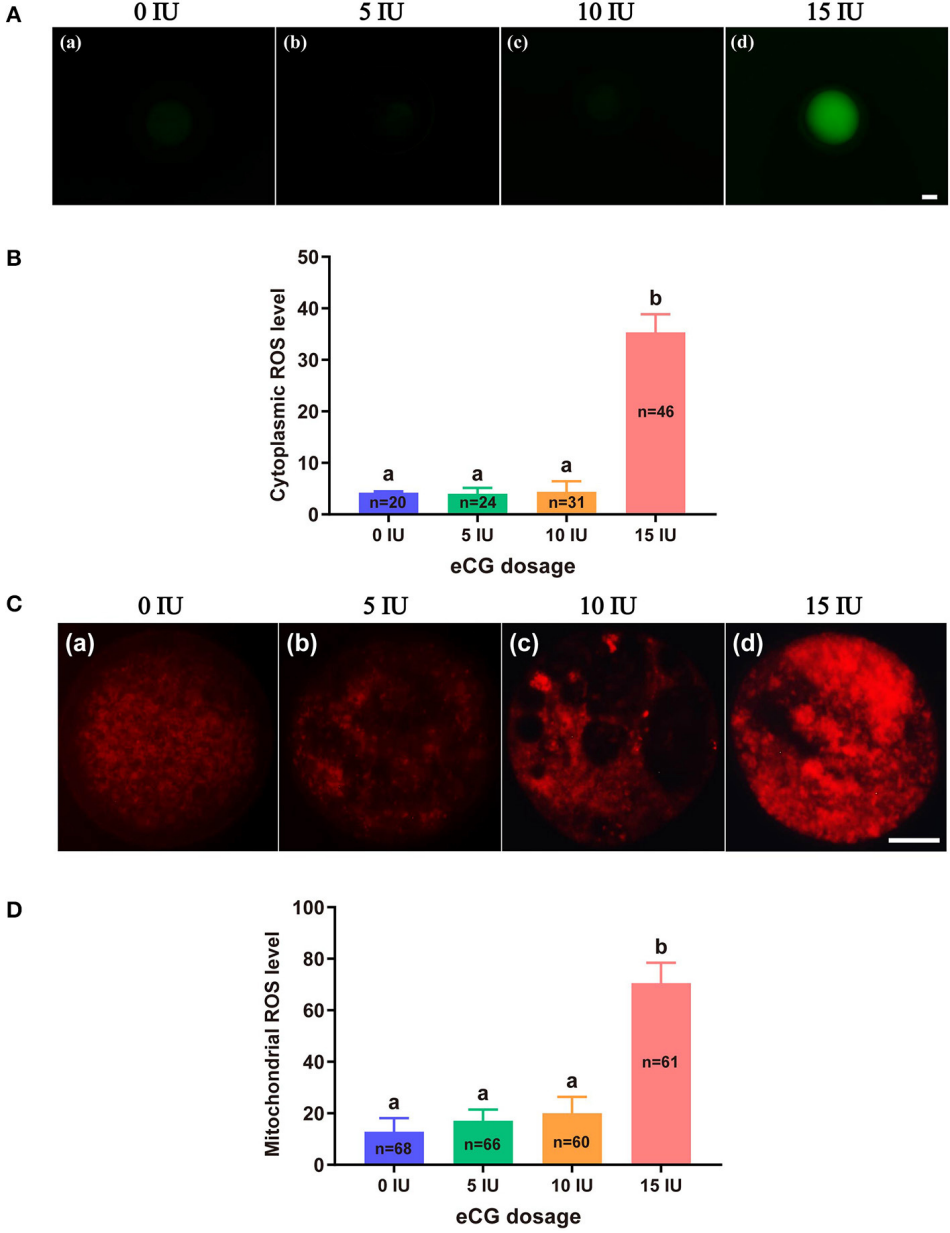
Effects of different eCG doses on ROS concentrations in IVF-derived mouse embryos. **(A)** Representative images of cytoplasmic ROS in embryos after administration of eCG at doses of 0, 5, 10, and 15 IU (a–d). Scale bar = 20 μm. **(B)** ROS production in the cytoplasm was assessed by staining with DCFH-DA. The cytoplasmic ROS levels of the embryos in the group treated with the high dose (15 IU) of eCG were significantly higher than those of the embryos in the other three groups. **(C)** Representative images of mitochondrial ROS in embryos after administration of eCG at doses of 0, 5, 10, and 15 IU (a–d). Scale bar = 20 μm. **(D)** ROS production in mitochondria was assessed using the mitochondrial superoxide indicator MitoSOX™ Red. The mitochondrial ROS levels of the embryos in the group treated with the high dose (15 IU) of eCG were significantly higher than those of the embryos in the other three groups. The continuous FI data were analyzed using Student's *t*-test and ANOVA. ^a,b^Bars labeled with different letters are significantly different from each other (*P* < 0.05). *n* shows the total number of embryos detected.

The total cytoplasmic and mitochondrial ROS levels of mouse embryos obtained after administration of different doses of eCG were tested using the cell-permeable fluorogenic probe DCFH-DA and the mitochondrial superoxide indicator MitoSOX™ Red, respectively. Our results showed that the total cytoplasmic ROS levels (35.35 ± 3.50, *P* < 0.001) and mitochondrial ROS levels (70.57 ± 7.88, *P* < 0.001) of the embryos in the 15 IU eCG group were markedly higher than those of the embryos in the other three groups. The mean FIs of cytoplasmic and mitochondrial ROS produced in the embryos of the 10 IU eCG treatment group (4.40 ± 2.04 and 20.01 ± 6.38, respectively) were similar to those in the embryos of the 5 IU eCG treatment group (4.04 ± 1.12 and 17.09 ± 4.40, respectively) and the control group (4.22 ± 0.25 and 12.88 ± 5.24, respectively) (Figure 2).

### Effects of Different eCG Doses on the Mitochondrial Function of IVF-Derived Mouse Embryos

To further verify the effects of different eCG doses on mitochondrial function in IVF mouse embryos, we monitored variations in MMP, active mitochondria, and ATP content.

#### Measurement of MMP

**Figure 3:**
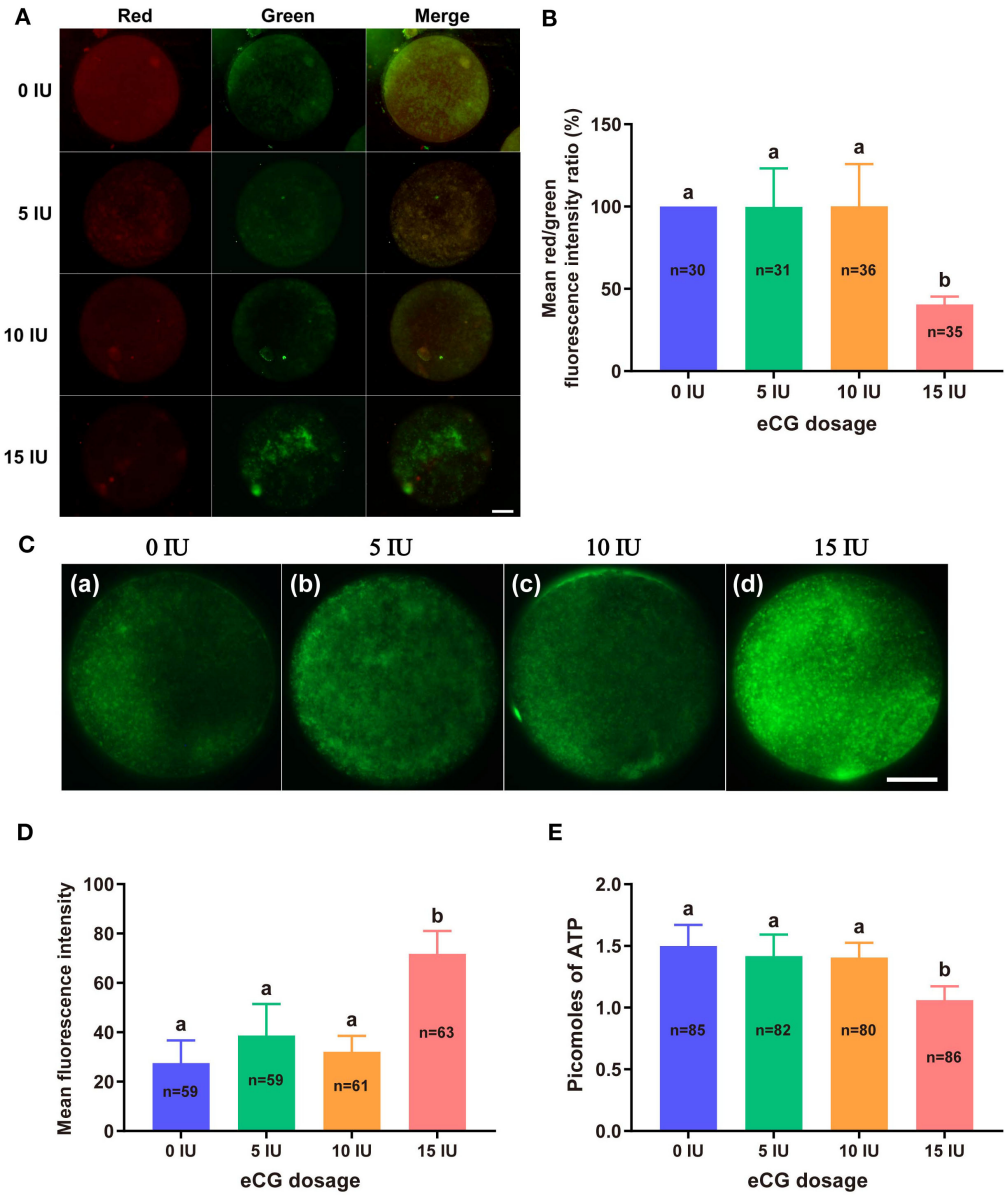
Effects of different eCG doses on the mitochondrial function of IVF-derived mouse embryos. **(A)** Representative images of the MMP in embryos after administration of eCG at doses of 0, 5, 10, and 15 IU. Scale bar = 20 μm. **(B)** Ratios of the red/green FI following JC-1 staining. The mean red/green FI ratio of the control (0 IU eCG injection) group was set to 100%. **(C)** Representative images of mitochondrial activity in embryos after administration of eCG at doses of 0, 5, 10, and 15 IU (a–d). Scale bar = 20 μm. **(D)** Magnitude of mitochondrial activity in IVF-derived embryos after administration of different doses of eCG. The mitochondrial activity in the embryos is represented by the mean FI. **(E)** ATP content of individual embryos from mice administered different doses of eCG. All the values are presented as the means ± *SD*s. Different letters above the columns indicate significant differences (*P* < 0.05). Each experiment was performed in triplicate, and *n* shows the total number of embryos detected.

JC-1 tends to aggregate and thus fluoresce red when MMP is elevated. In contrast, JC-1 tends to appear as a monomer, fluorescing green, when MMP is reduced. Thus, the FI ratio of these two colors of fluorescence (red/green ratio) is often used to evaluate changes in MMP. In this experiment, the mean red/green FI ratio obtained for the control group was set to 100%. No significant difference in MMP was found among the embryos from the 0, 5, and 10 IU eCG treatment groups. The relative red/green FI ratio for the embryos retrieved from the mice injected with 15 IU eCG was ~2.5-fold lower than that for the embryos retrieved from the control mice (100 vs. 40.58% ± 4.72, *P* < 0.001) (Figures 3A,B).

#### Evaluation of Active Mitochondria

We used MitoTracker™ Green to assess the number of active mitochondria, which is expressed as the FI per embryo. The embryos obtained from the mice injected with 15 IU eCG showed higher mitochondrial activity than the IVF-derived embryos in the other three groups (*P* < 0.001). Moreover, administration of eCG at doses of 0, 5, and 10 IU did not significantly affect the number of active mitochondria. Although the number of active mitochondria was slightly increased in the 5 IU eCG treatment group, it was very similar to those in the 0 and 10 IU eCG treatment groups (Figures 3C,D).

#### Quantification of ATP Content

A 14-point standard curve was generated in each experiment. The mean ATP content (1.06 ± 0.11, *P* < 0.05) in the IVF embryos obtained after injection of 15 IU eCG was lower than that in the embryos obtained from control mice. The mean ATP levels of the individual embryos obtained from the female mice administered 0, 5, and 10 IU eCG were 1.50 ± 0.17, 1.42 ± 0.18, and 1.41 ± 0.12, respectively, and no significant changes in ATP were detected in these three groups (*P* > 0.05) (Figure 3E).

### Effects of Different eCG Doses on Chromosome Segregation and the Cytoskeleton in IVF-Derived Mouse Embryos

To study the effects of different eCG doses on chromosome segregation and the cytoskeletal system in embryos obtained from IVF, we explored lagging chromosomes, micronuclei and multinuclei through DAPI staining; determined the number of chromosomes by karyotype analysis; and detected the localization of α-tubulin (microtubules) and F-actin (microfilaments) by immunostaining.

#### Determination of Lagging Chromosomes, Micronuclei, and Multinuclei

**Figure 4:**
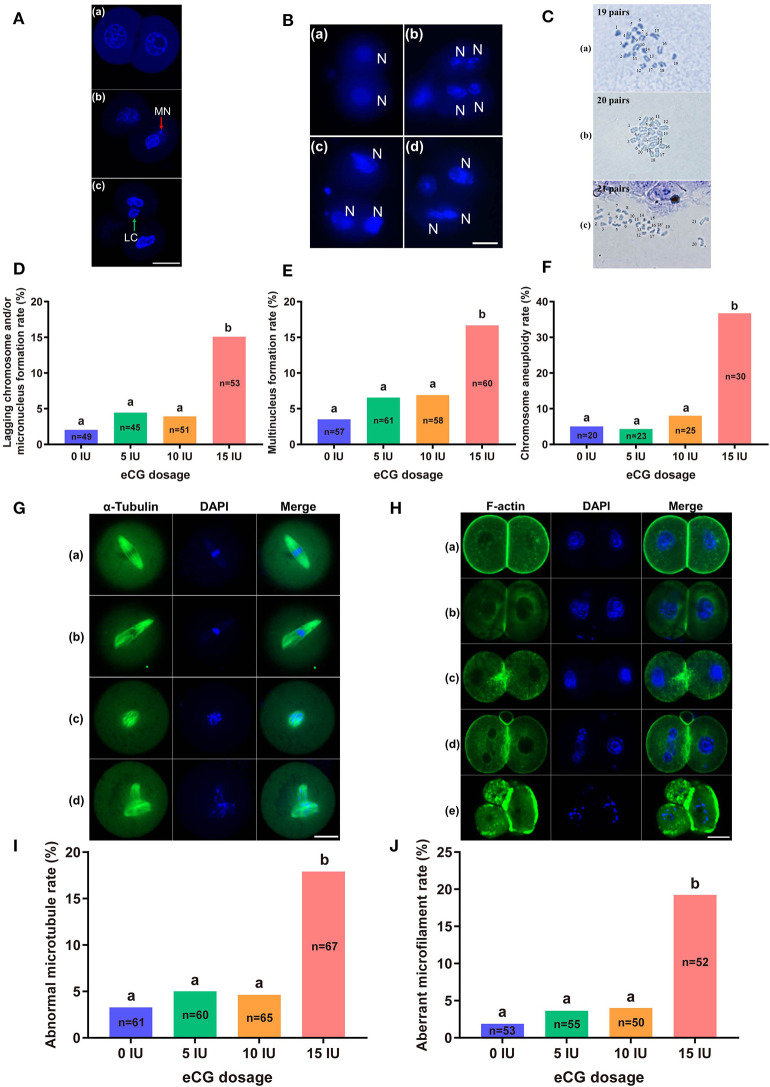
Effects of different eCG doses on chromosome segregation and the cytoskeleton in IVF-derived mouse embryos during the first mitotic division. **(A)** Fluorescence photomicrograph of two normal daughter nuclei, micronuclei (red arrow) and lagging chromosomes (green arrow) (a–c, respectively) in IVF-derived mouse embryos during the first mitosis. Scale bar = 20 μm. MN and LC refer to micronuclei and lagging chromosomes, respectively. **(B)** Representative images of two new daughter nuclei (a) and multinuclei (b–d). Scale bar = 20 μm. *N* refers to nuclei. **(C)** Hypodiploidy: 19 pairs of chromosomes (a). Normal mice have 20 pairs of chromosomes (b). Hyperdiploidy: 21 pairs of chromosomes (c). **(D–F)** Bar graphs of the lagging chromosome and/or micronucleus formation rates, multinucleus formation rates, and chromosome aneuploidy rates for the four groups. **(G)** Confocal images of a normal spindle structure and shape (a) and abnormal spindle formation (b–d). Mouse embryos at metaphase were stained with anti-α-tubulin antibodies (green) to detect microtubules, namely, the spindle. Scale bar = 20 μm. **(H)** Mouse embryos were stained with Alexa Fluor™ 488 phalloidin (green) to visualize the configuration and distribution of microfilaments. Normal microfilaments were precisely located in the cell cortex around the contractile ring at telophase (a). Representative images of aberrant microfilament formation (b–e). Scale bar = 20 μm. **(I,J)** Bar graphs of the abnormal microtubule rates and aberrant microfilament rates, respectively. Different lowercase letters in the columns within each parameter indicate significant differences (*P* < 0.05). The nuclei were stained with DAPI (blue), and *n* shows the total number of embryos detected.

The rates of lagging chromosome and/or micronucleus formation in IVF-derived embryos retrieved following administration of 0, 5, and 10 IU eCG were 2.04% (1/49), 4.44% (2/45), and 3.92% (2/51), respectively; these rates were significantly lower than that in the 15 IU eCG injection group (15.09%, 8/53, *P* < 0.05) (Figures 4A,D). A similar trend was observed for the rates of multinucleus formation in the various groups. The percentage of multinucleus formation among IVF-derived embryos from females treated with 5 (6.56%, 4/61) or 10 (6.90%, 4/58) IU eCG was comparable to that among embryos from control females (3.51%, 2/57) but significantly lower than that among embryos from females treated with 15 IU eCG (16.67%, 10/60) (Figures 4B,E).

#### Analysis of the Number of Chromosomes

Our chromosome karyotyping results suggested that the rates of chromosomal abnormalities (including hyperdiploidy and hypodiploidy) were significantly enhanced in IVF-derived embryos obtained with increasing doses of eCG up to 15 IU (36.67%, 11/30). Non-significant differences in the incidence of aneuploidy were observed among the groups of females injected with 0, 5, and 10 IU eCG; the rates of aneuploidy obtained for these groups were 5.00% (1/20), 4.35% (1/23), and 8.00% (2/25), respectively (Figures 4C,F).

#### Distribution of Microtubules and Microfilaments

The localization of α-tubulin (microtubules) is related to the formation of the spindle at the metaphase of mitosis. Importantly, the assembly and distribution of F-actin cytoskeletal components (microfilaments) are associated with cleavage in early-stage embryos. Similar trends in the rates of both abnormal microtubule formation and aberrant microfilament formation were found in all four groups. The rates of abnormal microtubule and aberrant microfilament formation in IVF-derived embryos obtained after ovarian stimulation with 15 IU eCG were 17.91% (12/67) and 19.23% (10/52), respectively; these values were substantially higher than those found for the embryos obtained after ovarian stimulation with 0 IU eCG (3.28%, 2/61 and 1.89%, 1/53, respectively), 5 IU eCG (5.00%, 3/60 and 3.64%, 2/55, respectively), and 10 IU eCG (4.62%, 3/65 and 4.00%, 2/50, respectively) (Figures 4G–J).

### Effects of Different eCG Doses on Apoptosis and Cell Allocation in IVF-Derived Mouse Blastocysts

**Figure 5:**
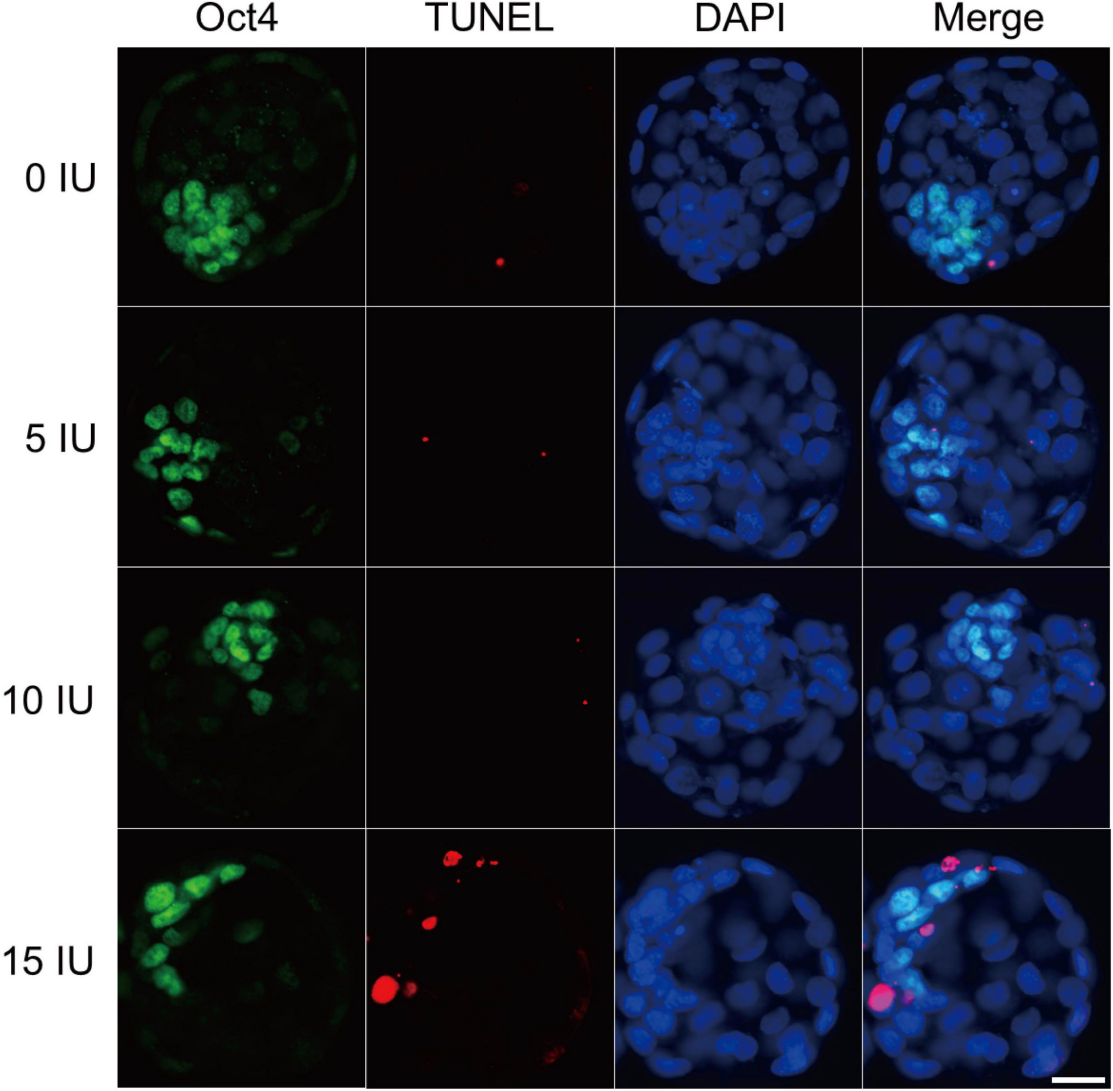
Effects of different eCG doses on apoptosis and cell allocation in IVF-derived mouse blastocysts. Representative fluorescence micrographs of IVF-derived blastocysts obtained from groups primed with different doses of eCG are shown. The proportion of ICM cells was inferred by counting Oct4-positive (green) cells. The percentage of apoptotic nuclei was examined by detecting TUNEL-positive (red) cells. DAPI staining (blue) was used to analyze the total number of nuclei and thus estimate the total cell count of individual blastocysts. Scale bar = 20 μm.

The developmental potential of IVF-derived blastocysts from groups of mice administered 0, 5, 10, and 15 IU eCG was evaluated with respect to apoptosis, total cell numbers and allocation of cells to the ICM and TE (Figure 5). As indicated in Table 1, increasing eCG doses up to 15 IU significantly enhanced the average apoptotic cell count and the mean apoptotic rate compared with the values obtained for IVF-derived blastocysts retrieved from mice primed with 0, 5, and 10 IU eCG (*P* < 0.05). The IVF-derived blastocysts in the 15 IU eCG treatment group had the lowest numbers of total cells, ICM cells, and TE cells and the lowest ICM/TE cell ratio (*P* < 0.05). Our results revealed that administration of 0, 5, and 10 IU eCG had no obvious effects on the total cell count, number of ICM cells, number of TE cells, or ICM/TE cell ratio in individual IVF-derived embryos, as shown in Table 2.

**Table 1:** Comparison of the average apoptotic cell counts and the mean apoptotic rates in IVF-derived blastocysts obtained from mice primed with different doses of eCG.

**eCG dose (IU)**	**Number of blastocysts examined**	**Total cell count**	**Average apoptotic cell count**	**Mean apoptotic rate (%)**
0	35	50.26^a^ ± 6.68	1.63^a^ ± 1.03	3.16^a^ ± 1.88
5	35	48.86^a^ ± 5.77	1.71^a^ ± 1.10	3.54^a^ ± 2.28
10	35	48.43^a^ ± 5.90	1.54^a^ ± 1.04	3.17^a^ ± 2.06
15	35	36.54^b^ ± 4.66	5.46^b^ ± 2.50	14.77^b^ ± 6.56

*The values are shown as the means ± SDs*.

a,b *Different superscripts within the same column indicate statistically significant differences (P < 0.05)*.

**Table 2:** Comparison of the allocation to ICM and TE lineages in IVF-derived blastocysts derived from mice primed with different doses of eCG.

**eCG dose (IU)**	**ICM cell count**	**TE cell count**	**ICM/TE cell ratio**
0	16.09^a^ ± 2.32	34.17^a^ ± 4.62	0.47^a^ ± 0.04
5	16.14^a^ ± 2.12	32.71^a^ ± 4.36	0.50^a^ ± 0.07
10	15.49^a^ ± 2.08	32.94^a^ ± 4.17	0.47^a^ ± 0.05
15	10.29^b^ ± 1.71	26.26^b^ ± 4.35	0.40^b^ ± 0.10

*The data are expressed as the means ± SDs*.

a,b *Within a column, values with different superscripts are significantly different (P < 0.05)*.

### Effects of Different eCG Doses on the Expression Levels of γH2AX, Aurora B, and MAD2L1 in IVF-Derived Mouse Embryos

We subsequently investigated the roles of the DNA damage response (DDR), CPC and SAC in the response of IVF-derived mouse embryos to different eCG doses. We examined both the subcellular localization and the relative expression levels of γH2AX (a marker of DNA double-strand breaks), Aurora B (the central member of the CPC), and MAD2L1 (a pivotal component of the SAC).

#### Immunofluorescence Staining for γH2AX, Aurora B, and MAD2L1

**Figure 6:**
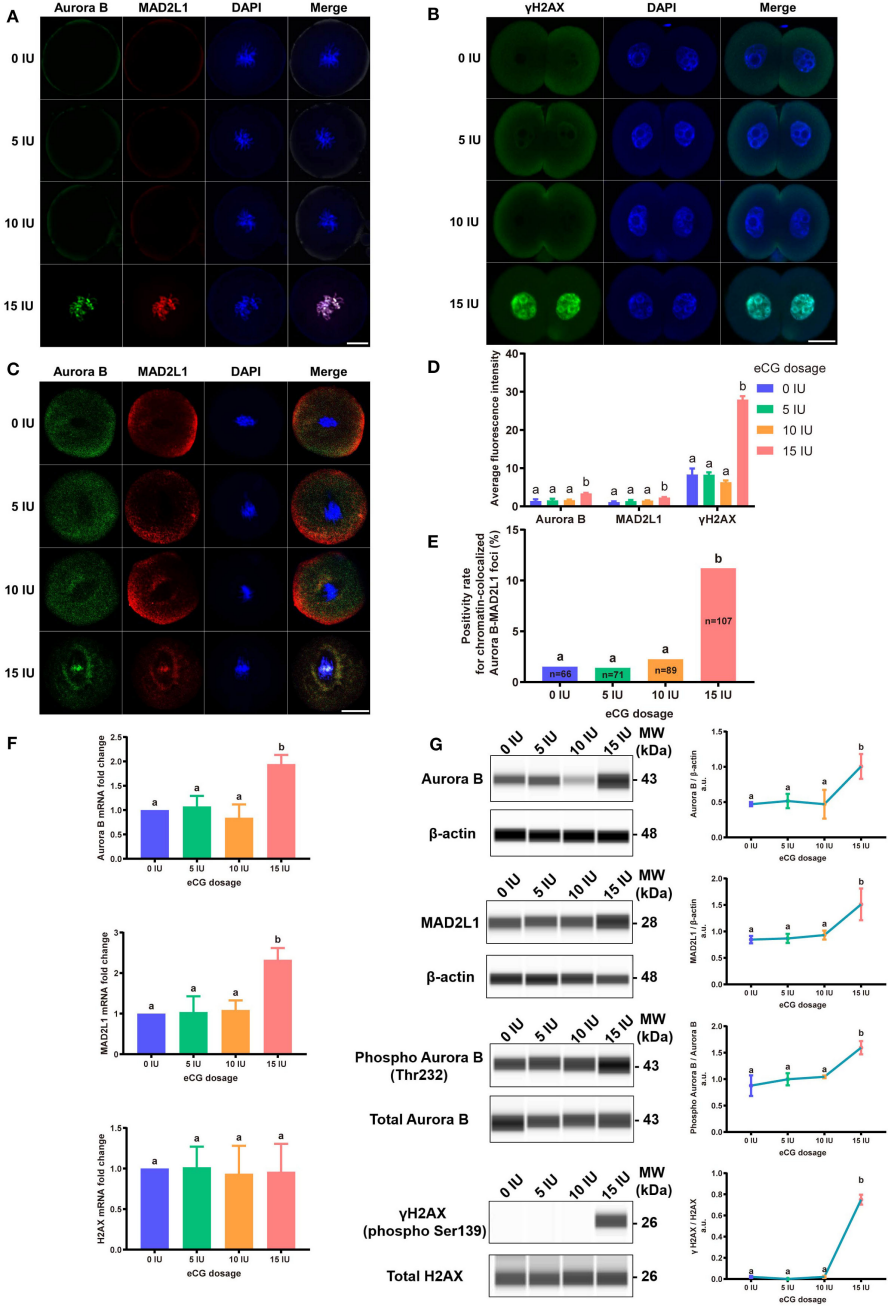
Effects of different eCG doses on the expression profiles of γH2AX, Aurora B, and MAD2L1 in IVF-derived mouse embryos. **(A)** The subcellular colocalization between Aurora B (green) and MAD2L1 (red) in IVF-derived mouse embryos from groups treated with different eCG doses was observed by immunofluorescence staining. The nuclei were stained with DAPI (blue). Scale bar = 20 μm. **(B)** Representative photomicrographs of γH2AX in each group. In the 15 IU eCG treatment group, γH2AX (green) was strikingly expressed in two daughter nuclei (blue). Scale bar = 20 μm. **(C)** Confocal images of RNA FISH for endogenous Aurora B (green) and MAD2L1 (red) mRNA molecules in IVF-derived mouse embryos following administration of different doses of eCG. The embryo nuclei were counterstained with DAPI (blue). Scale bar = 20 μm. **(D)** Average FI of Aurora B/MAD2L1/γH2AX per IVF-derived mouse embryo in the groups treated with different eCG doses. The continuous FI data were analyzed using Student's *t*-test and ANOVA. ^a,b^Different lowercase letters in the columns within each parameter indicate significant differences (*P* < 0.05). **(E)** Percentages of IVF-derived mouse embryos positive for local enrichment of Aurora B and MAD2L1 mRNA signals on chromatin (positivity rates for chromatin-colocalized Aurora B-MAD2L1 foci) in groups of mice administered eCG once at doses ranging from 0 to 15 IU. *n* shows the total number of embryos. Different letters in the columns depict statistical significance (for a and b, *P* < 0.05). **(F)** The relative mRNA expression levels of Aurora B, MAD2L1, and H2AX were analyzed by RT-PCR and normalized to those of β-actin. The differences in these continuous data were analyzed by Student's *t*-test and ANOVA. ^a,b^Columns marked with different letters are significantly different from each other (*P* < 0.05). **(G)** Capillary-based immunodetection of protein extracts was performed using a Wes capillary-based device (ProteinSimple) to determine the average levels of Aurora B, MAD2L1, phospho-Aurora B (T232), and γH2AX. Every experiment was performed in triplicate. ^a,b^Different superscripts above the error bars show significant differences (*P* < 0.05).

To explore the relative protein expression levels of γH2AX, Aurora B, and MAD2L1, we further assessed the average FI values in IVF-derived mouse embryos belonging to all four groups with Image-Pro Plus 6.0 software. The outcomes are presented as the FI values in arbitrary units (a.u.) per embryo. High γH2AX expression was detected in a pair of daughter nuclei in IVF-derived embryos obtained from mice administered 15 IU eCG (*P* < 0.01). The average FI values of γH2AX in the groups of mice administered 0, 5, 10, and 15 IU eCG were 8.36 ± 1.56, 8.30 ± 0.63, 6.34 ± 0.43, and 27.95 ± 0.91, respectively. Strong enrichment of Aurora B-MAD2L1 foci on chromatin was observed in the 15 IU eCG treatment group, but weak expression was observed in the 0, 5, and 10 IU eCG treatment groups. The average FI values of Aurora B/MAD2L1 per embryo obtained after administration of 0, 5, 10, and 15 IU eCG were 1.43 ± 0.43/1.12 ± 0.19, 1.58 ± 0.45/1.40 ± 0.27, 1.64 ± 0.15/1.50 ± 0.09, and 3.42 ± 0.13/2.33 ± 0.10, respectively (Figures 6A,B,D).

#### Analysis of the Localization of Aurora B and MAD2L1 by FISH Assay

Due to the varied expression levels of Aurora B protein in different mouse strains and to differences in detection reagents, the presence of Aurora B protein in a single embryo cannot always be reliably detected using commercially available reagents (Schindler et al., 2012). Few studies have reported data resulting from detection of Aurora B expression in embryos *via* RNA *in situ* hybridization. To investigate the sensitivity of the RNA-FISH assay in detecting the localization of Aurora B and MAD2L1, we analyzed the subcellular localization of the corresponding mRNA molecules in IVF-derived mouse embryos. The double RNA-FISH results showed that Aurora B and MAD2L1 mRNA signals were strongly colocalized on chromatin in 11.21% (12/107) of IVF embryos derived from mice primed with 15 IU eCG. In contrast, enrichment of Aurora B and MAD2L1 signals on chromatin was detected in only 1.52% (1/66), 1.41% (1/71), and 2.25% (2/89) of the IVF embryos from female mice injected with 0, 5, and 10 IU eCG, respectively. In most other cases, Aurora B and MAD2L1 mRNA signals were localized in the cytoplasm (Figures 6C,E).

#### RT-PCR and Quantitative Immunocapillary Electrophoresis Analysis

Consistently, both the mRNA and protein expression levels of Aurora B and MAD2L1 in mouse embryos were significantly enhanced with increasing eCG doses up to 15 IU. Additionally, the relative protein expression levels of γH2AX and phospho-Aurora B (p-Aurora B; Aurora B phosphorylated at T232 and exhibiting maximal activation) were significantly higher in the 15 IU eCG treatment group than in the other three groups (*P* < 0.05). However, the mRNA expression of H2AX was not changed regardless of the eCG dose used (*P* > 0.05) (Figures 6F,G).

## Discussion

Traditional IVF ovarian stimulation strategies aim to maximize oocyte production. Recently, mild superovulation treatment protocols using lower doses and/or shorter durations of exogenous gonadotropin treatment have become increasingly popular, and the improvements in IVF technology have reduced the need for high oocyte production (Alper and Fauser, 2017). eCG mimics endogenous FSH with regard to its oocyte maturation-inducing effect, as observed in a mouse study (Behringer et al., 2018). In addition, a recent study has shown that high-dose FSH treatment should be discontinued during ovarian stimulation with IVF in predicted low responders because high FSH doses might not increase the rate of live births and might cause harm to women undergoing IVF treatment (Leijdekkers et al., 2019). Furthermore, studies on laboratory animals and women undergoing IVF have indicated that high doses of gonadotropin have harmful effects compared with low doses of gonadotropin (Bosch et al., 2016), and exposure to elevated levels of gonadotropins after IVF treatment is considered a hazard factor for the development of ovarian tumors. In addition, the female reproductive system appears to be more susceptible to gonadotropin-related disorders than the male reproductive system (Rulli and Huhtaniemi, 2005). Nevertheless, the effects of high gonadotropin doses on IVF-derived embryos remain poorly understood (Alper and Fauser, 2017), particularly at the cellular level. Because previous studies on the use of gonadotropins have yielded contradictory and inconclusive results (Barash et al., 2017), we focused on IVF-derived embryos from eCG-primed mice to determine whether different doses of a single gonadotropin (eCG) affect the developmental competence of IVF-derived embryos and the formation of aneuploidy.

In this study, oocyte yield increased with increasing eCG doses; this result is similar to findings obtained previously (Edgar et al., 1987; Karagenc et al., 2004). Additionally, we observed that exposure of IVF-derived embryos to relatively high eCG doses reduced fertilization, four-cell embryo formation and blastocyst formation rates but did not significantly affect the two-cell embryo formation rate. Although we observed significant increases in oocyte yield in the presence of high doses of gonadotropin, the increases appeared meaningless because no effective increases in the numbers of high-quality embryos and blastocysts or in the live birth rate, which is the ultimate outcome, were observed (Leijdekkers et al., 2019). Undoubtedly, the success of IVF depends largely on the quality of the oocytes (Bosch et al., 2016). Notably, the development of diploid parthenogenetic oocytes is impaired by high eCG doses, which suggests that superovulation reduces both oocyte developmental potential and oocyte quality (Karagenc et al., 2004).

ROS can be derived from embryo metabolism and/or the environment surrounding embryos. Culture conditions and some other exogenous factors can accelerate the generation of ROS in embryos (Guérin et al., 2001). Oxidative stress plays a vital role in the process of ovulation, and ovulation leads to ROS-induced damage, which accumulates stage by stage in oocytes and related cells (Nie et al., 2018). We found higher total cytoplasmic and mitochondrial ROS levels in the embryos of the 15 IU eCG treatment group than in the embryos of the other groups, which indicated that IVF-derived embryos from mice primed with 15 IU eCG were in a state of oxidative stress. Ovarian stimulation during IVF can lead to supraphysiological estradiol concentrations, and elevated estradiol concentrations induce ROS production and mitochondrial dysfunction (Chou et al., 2020). In addition to genetic susceptibility factors, the quality of oocytes depends to a large extent on their maturation environment within the follicle and their independent viability after ovulation (Bradley and Swann, 2019). Superovulation increases the levels of ROS in the ovaries of mice, which demonstrates that ovaries are under high levels of oxidative stress after superovulation (Nie et al., 2018). Stimulation of the oviductal environment by gonadotropins impairs the development of embryos. Specifically, after superovulation, the presence of fluid in the fallopian tube appears to hinder embryo development (Chegini, 1996). Follicular fluid is the microenvironment of mature oocytes before fertilization, and the levels of ROS extracted from the follicular fluid of women undergoing ovarian stimulation is significantly negatively correlated with embryo formation and quality (Das et al., 2006). *In vitro* culture appears to compromise the development of IVF-derived embryos, particularly with respect to ROS levels, which indicates that non-ideal culture environments might be responsible for some of the observed results (Huang et al., 2019; Li et al., 2019) and that high doses of eCG may worsen these results.

It is important to consider that mitochondrial status and activity, which are susceptible to influence by non-physiological processes such as ovarian hyperstimulation and IVF, are key aspects affecting the quality of oocytes and the results of IVF (Ge et al., 2012). A mitochondrial ATP content above the threshold value of 2.0 pmol in human oocytes is connected with an increased likelihood of normal development and of early implantation of embryos after fertilization (Thouas et al., 2004). However, the ATP content and MMP of oocytes decrease after repeated ovarian stimulation (Combelles and Albertini, 2003; Ge et al., 2012). Hypoxia can increase MMP in mouse blastocysts cultured *in vitro* while elevating the expression levels of antioxidant genes and implantation proteins (Ma et al., 2017). In addition, MMP is involved in regulation of ROS and ATP generation (Romek et al., 2017), and a reduction in MMP is an early irreversible step in apoptosis (Zamzami et al., 1995; Green and Reed, 1998). The energy needed to maintain the normal development of preimplantation embryos is supplied in the form of ATP produced by mitochondria. Studies have shown that a suboptimal level of mitochondrial ATP production might lead to multiple embryo development defects and thus adversely affect the success rate of IVF (Fragouli and Wells, 2015). In this study, increasing doses of eCG decreased MMP and sharply reduced the ATP content. In addition, the highest mitochondrial activity was obtained in the 15 IU eCG treatment group. Our findings are in good agreement with the “quiet embryo hypothesis” presented by Baumann et al. (2007) and Jing et al. (2019), who proposed that the active metabolism observed in embryos prior to the blastocyst stage is a signal that triggers multiple energy-consuming pathways for DNA damage repair. The viability of early embryos depends on their ability to maintain optimal metabolic levels (Bradley and Swann, 2019). Compared with less metabolically active embryos, embryos with more active metabolism show greater damage, and the surviving embryos exhibit lower oxidative phosphorylation activity and reduced oxygen consumption (Madrid Gaviria et al., 2019). The metabolism of an embryo before implantation is related to its developmental potential (Uyar and Seli, 2014). Quiet metabolism in early-stage embryos is associated with well-balanced embryo development. In contrast, active metabolism is related to subsequent suboptimal developmental outcomes (Leese et al., 2007). When external factors affect the viability of embryos, energy consumption increases to enable completion of the necessary repair processes. Active metabolism increases ROS levels with potentially harmful outcomes for embryos (Leese et al., 2007). Relatively low metabolic levels are most conducive to embryo survival (Leese, 2002). Notably, embryos cultured in 20% oxygen are capable of development and indeed exhibit reduced nutrient turnover (Leese et al., 2008). Suboptimal embryos cannot tolerate oxidative stress. Developmentally impaired or unhealthy embryos might have weakened defenses or reduced antioxidant capacities to cope with ROS and might therefore have to resort to other energy-consuming metabolic processes to maintain their redox stability. The quiet embryo hypothesis in this case refers to the ability of an embryo to respond to ROS (Gardner and Wale, 2013). Healthy embryos are metabolically inactive. Any non-physiological conditions that impair the function of an embryo trigger active metabolism, which may be reflected by an enhanced expenditure of energy substrates from the surrounding environment (D'Souza et al., 2016). Conceivably, to compensate for the oxidative damage caused by high-dose eCG-mediated induction of ovulation, the embryos in the current study underwent an energy-consuming repair process, which might explain the increases in active mitochondria and mitochondrial ROS production and the decreases in MMP and ATP content observed in the IVF mouse embryos belonging to the 15 IU eCG treatment group.

Sudden increases in the levels of blood gonadotropins (endogenous or exogenous) are potential mechanisms of aneuploidy (Dursun et al., 2006). In addition, gonadotropins might exert a mutagenic effect on DNA and increase sister chromatid exchange rates (Dursun et al., 2006). During the eight to sixteen-cell embryo stage, the dose of eCG and the probability of polyploidy show a dose-response relationship in CD-1 mice, and the probability of polyploidy increases from 2.9% with 10 IU eCG to 10.5% with 15 IU eCG (Ma et al., 1997). In our previous model, we revealed that ROS-induced damage increases the incidence of sex chromosome aneuploidy in IVF-obtained male mouse embryos and found that this effect occurs mainly *via* chromosome mis-segregation (Huang et al., 2019). The frequent observation of lagging chromosomes, micronuclei, and/or multinuclei suggests the existence of chromosomal aneuploidy (Zhao et al., 2010; Huang et al., 2019; Li et al., 2019). The formation of micronuclei in mouse embryos leads to permanent unilateral chromosomal inheritance (Vázquez-Diez et al., 2016). Our results demonstrate that a high gonadotropin dose leads to an elevated incidence of nuclear abnormalities; in other words, high-dose gonadotropin stimulation might induce chromosomal aneuploidy.

The side effects of exogenous gonadotropins may include cytoskeletal abnormalities. For example, spindle assembly and chromosome segregation might be affected by ovarian hormone stimulation (Mantikou et al., 2012), and abnormal spindle formation usually leads to aneuploidy (Zhao et al., 2010). Gonadotropins may also influence microtubule function in meiotic and mitotic spindles and thus increase aneuploidy rates (Dursun et al., 2006). Moreover, the correct assembly and distribution of the microfilament cytoskeleton in mouse embryos are closely related to the cleavage of early embryos (Wu et al., 2016). Different modes of microfilament formation have been found in the oocytes of superovulated hamsters (Lee et al., 2005). High doses of gonadotropins can induce aberrant microfilament distribution and decrease enrichment of cortical actin domains in oocytes, which might be associated with abnormal expression of actin-related genes (Lee et al., 2006). These findings, in conjunction with the findings of the current study, provide evidence showing that high-dose stimulation exerts negative effects on the spindle structure and microfilament distribution in IVF-derived mouse embryos.

The blastocyst stage is the optimal time period for screening of abnormalities (Bazrgar et al., 2013). The quality of blastocysts depends on the number of cells in the ICM, the number of TUNEL-positive nuclei, the ratio of ICM cells to TE cells, and the total number of cells (Wang et al., 2006; Maluf et al., 2009; Romek et al., 2017). Oct4-expressing ICM and TE cells are separated during blastocyst formation (Le Bin et al., 2014; Simmet et al., 2018). Compared with *in vivo* fertilization, IVF negatively affects the number of TE cells and ICM cells in blastocysts (Maluf et al., 2009). Additionally, compared with blastocysts obtained from naturally cycling mice, blastocysts obtained from superovulated mice present fewer surface microvilli, and the decrease in surface microvilli is accompanied by decreases in [^35^S]-methionine uptake, cell number, mitotic index, and viability (Mitwally et al., 2005). According to studies conducted by Lee et al. (2005) the number of ICM cells is significantly reduced in golden hamsters after superovulation, and this reduction might be linked with alterations in mitochondrial function or number. One early study performed by our research team indicated that ROS increase the blastocyst apoptosis rate (Huang et al., 2019). A major consequence of unrepaired DNA damage is apoptosis. Mechanistically, it is likely that DNA damage causes developmental delay and micronuclei in embryos and ultimately leads to apoptosis in blastocysts. Normal embryos with DNA damage show increased genomic instability, which might affect implantation and postimplantation development (D'Souza et al., 2016). Notably, the total cell counts and ICM and TE cell counts of diploid parthenogenetic blastocysts are significantly lower after application of 20 IU eCG than after application of 5 and 10 IU eCG (Karagenc et al., 2004). Similar to the results obtained with diploid parthenogenetic mouse blastocysts, rapid declines in the TE cell and ICM cell proportions, the ICM/TE cell ratios and the total cell counts in IVF-derived blastocysts were observed after stimulation with high doses of gonadotropin in this study.

Several hypotheses can be raised to explain the observed reductions in the developmental capacity of embryos in response to high doses of eCG. (1) Stimulation with high concentrations of eCG might have disrupted normal and critical intracellular signal transduction pathways that play crucial roles in the earliest phases of embryogenesis (Williams, 2002; Karagenc et al., 2004; Ducibella and Fissore, 2008). Gonadotropin-dependent follicles have their own microenvironments that transmit gonadotropin signals in different ways (Webb and Campbell, 2007). Tightly regulated signal transduction is important for embryonic functions (Lin et al., 2015), and high doses of eCG might disrupt the functions of normal signal transduction pathways at the early stages of development (Karagenc et al., 2004). (2) The administration of elevated levels of eCG for ovarian stimulation might have changed the levels and distributions of regulatory proteins in oocytes/embryos (Karagenc et al., 2004). (3) High-dose eCG treatment might have led to abnormalities in cytoskeletal dynamics and activity to negatively influence embryo proliferation and differentiation (Lee et al., 2005, 2006). (4) The high doses of eCG might have altered the mitochondrial number and activity in oocytes/embryos and induced mitochondrial dysfunction, resulting in insufficient energy or high oxidative stress (Lee et al., 2006). (5) The administration of high doses of gonadotropin might have induced production of a large quantity of low-potential oocytes. Low-potential oocytes are usually immature, with low mitochondrial counts and mitochondrial dysfunction, which lead to reduced rates of embryo development; such embryos might have been selected during the processes of fertilization and cleavage (Shu et al., 2016).

MAD2L1, a crucial component of the SAC complex, ensures chromosomal stability by regulating Aurora B (Shandilya et al., 2016). In a previous study, we first confirmed that ROS can arrest IVF-obtained mouse embryos in prophase/metaphase at the first mitotic cleavage through MAD2L1-mediated SAC activation (Wu et al., 2017). We then illustrated that SAC and DDR assist in repairing sex chromosome aneuploidy through the MAD2-mediated pathway (Huang et al., 2019). Subsequently, and most importantly, we demonstrated that Aurora B prevents aneuploidy *via* MAD2 in IVF-derived mouse embryos under oxidative stress (Li et al., 2019). In this study, we found that the expression of γH2AX was enhanced after administration of 15 IU eCG and that the expression of this protein remained restricted to two daughter nuclei at telophase in IVF-derived embryos. Furthermore, we detected distinctly upregulated expression and chromosomal enrichment of Aurora B and MAD2L1 in IVF-derived mouse embryos belonging to the high-stimulation (15 IU eCG) group. These results, combined with the research findings of our previous studies (Huang et al., 2019; Li et al., 2019) and the phenomenon of ROS elevation after high-dose stimulation, indicate that high-dose gonadotropin treatment induces ROS-mediated DNA damage and thereby triggers Aurora B-mediated SAC activation in early IVF-derived mouse embryos. Notably, it has previously been reported that partially activated Aurora B-mediated phosphorylation of H2AX at serine 121 (H2AX-pS121) can accelerate Aurora B full autophosphorylation, which is necessary for correct chromosome segregation in mitosis (Shimada and Nakanishi, 2016; Shimada et al., 2016). Given this information and our observations, we suspect that γH2AX plays a role similar to that of H2AX-pS121, but more experiments should be performed to confirm or refute this possibility. Further analysis *via* double mRNA FISH revealed that stimulation with 15 IU eCG increased the frequency of colocalization of Aurora B and MAD2L1 mRNA expression with chromatin in IVF-derived mouse embryos. The increased colocalization of these two mRNA populations with chromatin may be a sign of SAC activation. Our preliminary findings regarding Aurora B and MAD2L1 expression detected by mRNA FISH were similar to the immunofluorescence staining results. Due to the occasional failure of commercially available reagents to reliably detect Aurora B protein in individual oocytes and early embryos (Schindler et al., 2012; Nguyen and Schindler, 2017), mRNA FISH might be a more sensitive method than immunofluorescence for detecting Aurora B expression in IVF embryos. Findings obtained in mouse and Xenopus oocytes are in good agreement with our experimental results, suggesting that Aurora B may be detectable on all chromosomes during prometaphase (Ju et al., 2016). Moreover, Aurora B overexpression might partly rescue defects in chromosome alignment in mouse oocytes, specifically during the metaphase I stage (Shuda et al., 2009). Aurora B is also overexpressed in many tumors; however, this overexpression rarely leads to fatal aneuploidy because it limits chromosome segregation errors (Manzione et al., 2020). Aurora B enrichment on chromatin can also promote Aurora B activation (Kelly et al., 2007). Furthermore, estrogen induces the activity of Aurora B (Ruiz-Cortés et al., 2005). Notably, maximal Aurora B activation requires autophosphorylation at T232 in the activation loop (Shimada and Nakanishi, 2016). The capillary electrophoresis and immunodetection findings suggested that autophosphorylation of Aurora B at T232 was markedly strengthened by superovulation with 15 IU eCG, which supports the idea that Aurora B autoactivation is connected with SAC activation in IVF-derived embryos following administration of high doses of gonadotropin. The elevations in Aurora B and Aurora B autoactivation-triggered SAC activation (as indicated by increased expression of MAD2L1) seem designed to provide embryos with the chance to self-correct aneuploidies and repair their spindles during early embryo development (Wang et al., 2002; Bazrgar et al., 2013). Although chromosome instability is prevalent in cleavage-stage embryos during early IVF embryogenesis, mosaic embryos, including normal blastomeres, may still have the potential to be chromosomally normal fetuses (Vanneste et al., 2009). Of interest, some authors have reported that Aurora B p.L39P is a possible gain-of-function mutant protein with enhanced function in regulating the alignment of chromosomes at the metaphase plate (Nguyen et al., 2017). The expression and role of Aurora B in regeneration (Gwee et al., 2018; Shaalan and Proctor, 2019) and glycolysis metabolism (Zhou et al., 2018; He et al., 2019) have been explored over the past few years. Accordingly, the potential role of Aurora B in IVF-derived mouse embryos needs to be further confirmed and investigated.

**Figure 7:**
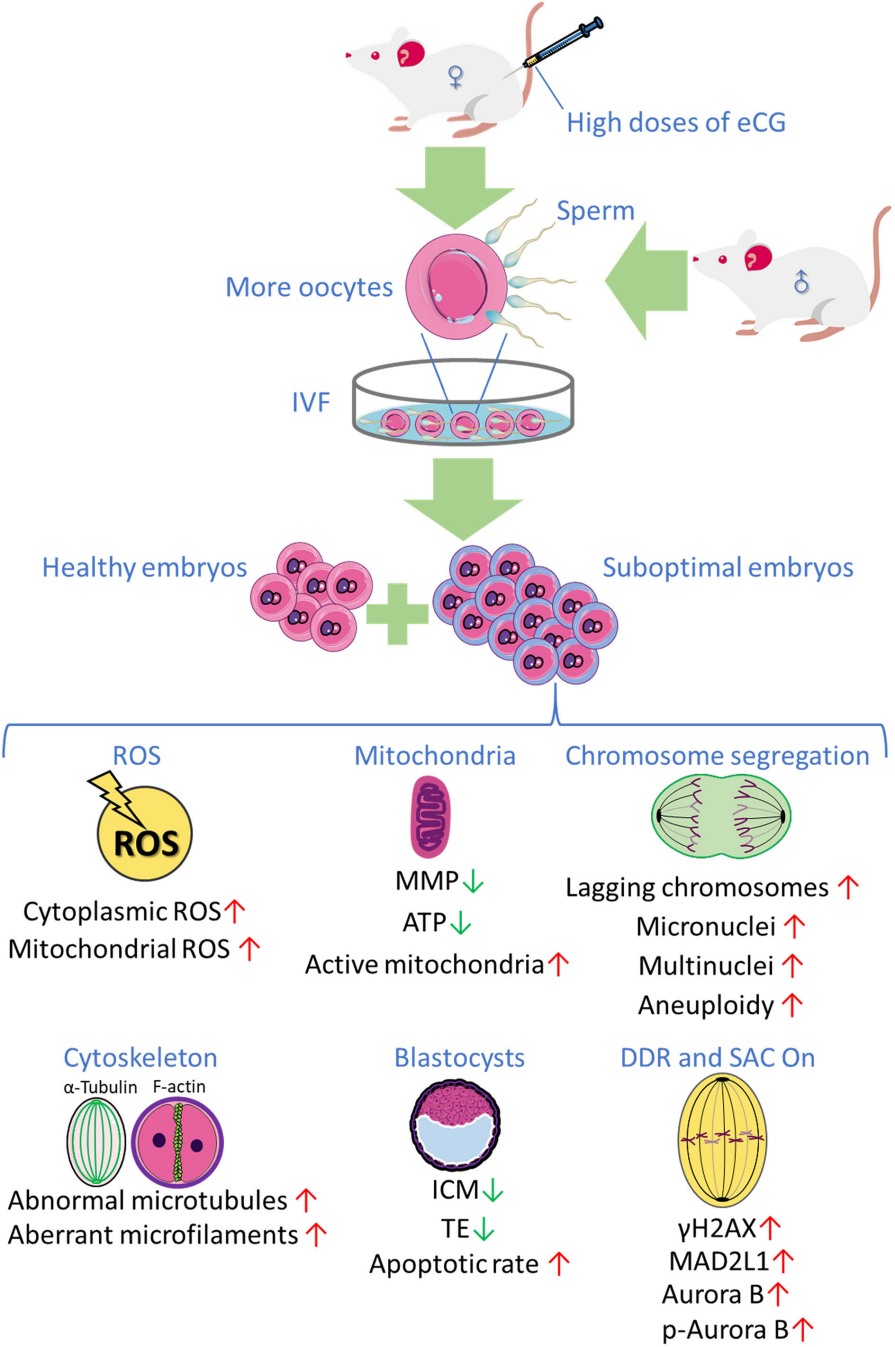
The research findings obtained in the present study reveal that priming with high doses of eCG exerts marked adverse effects on certain developmental competences of IVF-derived mouse embryos.

Collectively, the research findings obtained in the present study reveal that priming with high doses of eCG exerts marked adverse effects on certain developmental competences of IVF-derived mouse embryos. Based on these findings and the findings of our previous studies, we propose, for the first time, that a high dose of eCG is a contributing factor that might induce oxidative stress-related DNA damage to trigger Aurora B-mediated SAC activation in early IVF-derived mouse embryos for the self-correction of aneuploidy formation (Figure 7). Based on these considerations, the Aurora B protein and related agents likely play roles in preventing the birth of offspring with chromosomal diseases. It should be noted that this study examined only a mouse model, and there were some techniques and methods that we could not include; thus, the results might not be fully applicable to human ART, and suitable trials are needed to verify the findings. However, the lessons learned in studies on mice provide considerable evidence regarding the impact of gonadotrophin-induced superovulation on the developmental competence of IVF-derived embryos and its relationship with reproductive pathophysiology. The body surface area normalization method can be used to convert doses from mice to humans, especially in clinical trials (Reagan-Shaw et al., 2008). In the future, physiologic, pharmacokinetic, and toxicology data can also be used for scientific justification (Blanchard and Smoliga, 2015). Studies on mouse models have provided abundant new information on previously unknown mechanisms involved in the pathophysiological effects of gonadotrophins in both sexes and have clearly expanded the body of knowledge (Rulli and Huhtaniemi, 2005). In addition, new ART regimens involving low gonadotropin doses might be explored to improve pregnancy outcomes in women and to potentially avoid chromosome aneuploidy in embryos.

## Data Availability Statement

All datasets generated for this study are included in the article/Supplementary Material.

## Ethics Statement

The animal study was reviewed and approved by the Institutional Animal Care and Use Committee (IACUC) of Shantou University Medical College (SUMC2018-049).

## Author Contributions

ZL had the initial idea, supervised the experiments, and revised the manuscript. ZL, EL, and YH designed the experiments. EL, YH, GR, and PH performed the experiments and analyzed data. EL wrote the manuscript. All authors commented on the manuscript.

## Conflict of Interest

The authors declare that the research was conducted in the absence of any commercial or financial relationships that could be construed as a potential conflict of interest.
