# Supplementary material for: High Dosages of Equine Chorionic Gonadotropin Exert Adverse Effects on the Developmental Competence of IVF-Derived Mouse Embryos and Cause Oxidative Stress-Induced Aneuploidy
Source: Front Cell Dev Biol. 2021 Feb 9;8:609290. doi: 10.3389/fcell.2020.609290 (PMC7900142; doi:10.3389/fcell.2020.609290)
Supplement: Supplementary file 1 [file Data_Sheet_1.PDF]

Supplementary Material

1 Supplementary Table

Supplementary Table 1. The following primers were used in the amplification:

Gene	Sequence
β-actin	F：5’ ATGCCCTGAGGCTCTTTTCC 3’
	R：5’ ACGCGACCATCCTCCTCTTA 3’
Aurora B	F：5’ TGGAGAATGGCTCAGAAGGAG 3’
	R：5’ TGTTGGGATGTTTCAGGTGCG 3’
MAD2L1	F：5’ TCTTTGTCAGTGTAAATCAGCAG 3’
	R：5’ TCAGAAACTGGTGGTGGTC 3’
H2AX	F：5’ GGTAAGCGTCTCTCTGACCC 3’
	R：5’ CATACCAGTTGACCCTGTCG 3’
